# *catena*-Poly[[diaqua­bis­(3-methyl­pyridine-κ*N*)cobalt(II)]-μ-sulfato-κ^2^ *O*:*O*′]

**DOI:** 10.1107/S1600536811025815

**Published:** 2011-07-09

**Authors:** Naveed Alam, Matthias Zeller, Nur Syamimi Ahmad Tajidi, Zainudin Arifin, Muhammad Mazhar

**Affiliations:** a Department of Chemistry, Quaid-I-Azam University, Islamabad 45320, Pakistan; b Department of Chemistry, Youngstown State University, 1 University Plaza, Youngstown, Ohio 44555, USA; c Department of Chemistry, Faculty of Science, University of Malaya, Lembah Pantai, 50603 Kuala Lumpur, Malaysia

## Abstract

The environment of the Co^II^ ion in the title compound, [Co(SO_4_)(C_6_H_7_N)_2_(H_2_O)_2_]_*n*_, exhibits an octa­hedral configuration with the two 3-methyl­pyridine ligands lying in *cis* positions with respect to each other and *trans* to the two coordinated water mol­ecules. The axial positions are occupied by O atoms of the sulfate ions. Co and S atoms occupy special positions (twofold axis, Wyckoff position 4*c*). Neighboring Co^II^ ions are covalently connected with each other through the sulfate ions, thus creating infinite polymeric chains that run along the *c* axis. The water mol­ecules are connected with neighboring sulfate ions through strong O—H⋯O hydrogen bonds. Intra­molecular hydrogen bonds parallel to the propagation direction of the chains stabilize the polymeric chains, and inter­molecular hydrogen bonds between chains connect neighboring chains with each other, thus leading to polymeric double chains.

## Related literature

For the complexation of cobalt ions by sulfate, see: Das *et al.* (2009 ▶); Majumder *et al.* (2005 ▶); Masuhara *et al.* (2007 ▶); Zhong *et al.* (2006 ▶); Zhong *et al.* (2011 ▶); Dietz *et al.* (2009 ▶); Wu *et al.* (2008 ▶); Carlucci *et al.* (2003 ▶); Ali *et al.* (2005 ▶); Vreshch *et al.* (2003 ▶).
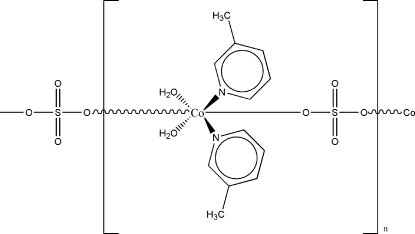


## Experimental

### 

#### Crystal data

[Co(SO_4_)(C_6_H_7_N)_2_(H_2_O)_2_]
*M* *_r_* = 377.27
Orthorhombic, 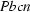

*a* = 15.132 (2) Å
*b* = 16.687 (2) Å
*c* = 6.4503 (9) Å
*V* = 1628.7 (4) Å^3^
*Z* = 4
Mo *K*α radiation
μ = 1.21 mm^−1^
*T* = 100 K
0.60 × 0.12 × 0.12 mm

#### Data collection

Bruker SMART APEX CCD diffractometer
Absorption correction: multi-scan (*SADABS*; Bruker, 2003 ▶) *T*_min_ = 0.786, *T*_max_ = 0.865
15656 measured reflections
2028 independent reflections
1892 reflections with *I* > 2σ(*I*)
*R*_int_ = 0.034

#### Refinement

*R*[*F* ^2^ > 2σ(*F* ^2^)] = 0.027
*wR*(*F* ^2^) = 0.075
*S* = 1.08
2028 reflections
108 parameters
2 restraints
H atoms treated by a mixture of independent and constrained refinement
Δρ_max_ = 0.57 e Å^−3^
Δρ_min_ = −0.32 e Å^−3^

### 

Data collection: *SMART* (Bruker, 2002 ▶); cell refinement: *SAINT-Plus* (Bruker, 2003 ▶); data reduction: *SAINT-Plus*; program(s) used to solve structure: *SHELXTL* (Sheldrick, 2008 ▶); program(s) used to refine structure: *SHELXTL*; molecular graphics: *Mercury* (Macrae *et al.*, 2008 ▶); software used to prepare material for publication: *publCIF* (Westrip, 2010 ▶).

## Supplementary Material

Crystal structure: contains datablock(s) I, global. DOI: 10.1107/S1600536811025815/fi2108sup1.cif

Structure factors: contains datablock(s) I. DOI: 10.1107/S1600536811025815/fi2108Isup2.hkl

Additional supplementary materials:  crystallographic information; 3D view; checkCIF report

## Figures and Tables

**Table 1:** Hydrogen-bond geometry (Å, °)

*D*—H⋯*A*	*D*—H	H⋯*A*	*D*⋯*A*	*D*—H⋯*A*
O1—H1*B*⋯O3^i^	0.82 (2)	1.86 (2)	2.6652 (15)	166 (2)
O1—H1*A*⋯O3^ii^	0.84 (2)	1.92 (2)	2.7331 (14)	165 (2)

Symmetry codes: (i) 
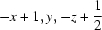
; (ii) 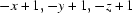.

**Fig. 1.**
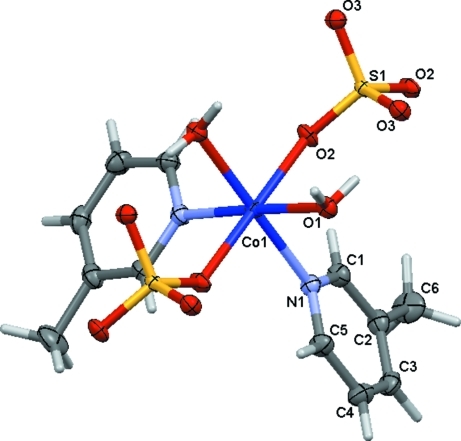
Thermal ellipsoid representation of the title compound with atom numbering scheme. Displacement elliposoids are at the 50% level, hydrogen atoms are shown as spheres of arbitrari radii. Symmetry operators: (i) -x + 1, y, -z + 1/2; (ii) -x + 1, y, -z + 3/2.

**Fig. 2.**
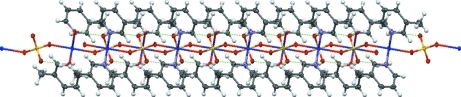
One of the infinite double chains formed by the title compound. View down the b-axis. O—H···O hydrogen bonds are symbolized as blue dashed lines.

**Fig. 3.**
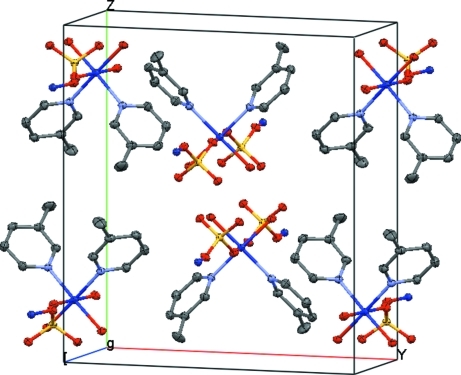
Packing arrangement of the title compound. H atoms have been omitted for clarity.

## supplementary crystallographic information

### Comment

Sulfate coordination to cobalt ions may be divided into three commonly reported modes: monodentate (Das *et al.*,2009, Majumder *et al.*, 2005), bidentate (Masuhara *et al.*., 2007, Zhong *et al.*,2006, 2011) or bidentate-bridged metal to metal coordination (Dietz *et al.*, 2009, Wu *et al.*, 2008, Carlucci *et al.*, 2003, Ali *et al.*, 2005, Vreshch *et al.*, 2003). The last mode of coordination is particularly common where the sulfate ion acts as a bridge that links two cobalt ions to form an extended polymeric structure. Further evidence for the different modes of sulfate coordination is reflected in the infra-red absorption spectrum due to the reduction in symmetry in sulfate coordination.

In the title compound, the cobalt(II) complex exhibits octahedral symmetry with the two 3-methylpyridine ligands lying in *cis* position with respect to each other, and *trans* to the two coordinated water molecules. The axial positions are occupied by oxygen atoms of the sulfate ions. Both Co and S occupy special positions (two-fold axis, Wyckoff position 4c). Neighboring cobalt ions are covalently connected with each other through the sulfate ions thus creating infinite polymeric chains that stretch parallel to the *c* axis direction. The water molecules are connected with neighboring sulfate ions through strong O—H···O hydrogen bonds. Intramolecular hydrogen bonds parallel to the propagation direction of the chains stabilize the polymeric chains, and intermolecular hydrogen bonds between chains connect neighboring strains with each other, thus leading to polymeric double chains.

### Experimental

Potassium *O*-*n*-butyl xanthate (1.00 g, 0.53 mmol) was dissolved in acetone (20 mL) and placed in a three-necked round bottom flask fitted with a reflux condenser, a magnetic stirrer and a vacuum line. Co(NO_3_)_2_.6H_2_O (0.78 g, 2.70 mmol) was added directly into the reaction flask. The contents were stirred to dissolve the salt completely. About 30 ml of 3-methylpyridine was added and stirring was continued for another hour. Any insoluble matter was removed by filtration, and slow evaporation of the reaction mixture at room temperature yielded 60% of red needles of the title compound as the unexpected product. m.p. = 373 K. Elemental analysis: Found (Calc.) for C_12_H_18_N_2_CoO_6_S: C 38.64 (38.20); H 4.66 (4.80); N 7.51 (7.42).

### Refinement

Water hydrogen atoms were located in the difference density Fourier map and their position were refined with an O–H distance restraint of 0.84 Å within a standard deviation of 0.02 Å. All other hydrogen atoms were placed in calculated positions and all H atoms were refined riding on the respective carrier atom with an isotropic displacement parameter 1.5 (methyl, hydroxyl) or 1.2 times (aromatic) that of the adjacent carbon or oxygen atom.

### Crystal data

**Table:**

[Co(SO_4_)(C_6_H_7_N)_2_(H_2_O)_2_]	*D*_x_ = 1.539 Mg m^−^^3^
*M**_r_* = 377.27	Melting point: 373 K
Orthorhombic, *P**b**c**n*	Mo *K*α radiation, λ = 0.71073 Å
Hall symbol: -P 2n 2ab	Cell parameters from 5623 reflections
*a* = 15.132 (2) Å	θ = 2.4–30.5°
*b* = 16.687 (2) Å	µ = 1.21 mm^−^^1^
*c* = 6.4503 (9) Å	*T* = 100 K
*V* = 1628.7 (4) Å^3^	Needle, red
*Z* = 4	0.60 × 0.12 × 0.12 mm
*F*(000) = 780	

### Data collection

**Table:**

Bruker SMART APEX CCD diffractometer	2028 independent reflections
Radiation source: fine-focus sealed tube	1892 reflections with *I* > 2σ(*I*)
graphite	*R*_int_ = 0.034
ω scans	θ_max_ = 28.3°, θ_min_ = 1.8°
Absorption correction: multi-scan (*SADABS* in *SAINT-Plus*; Bruker, 2003)	*h* = −20→19
*T*_min_ = 0.786, *T*_max_ = 0.865	*k* = −22→22
15656 measured reflections	*l* = −8→8

### Refinement

**Table:**

Refinement on *F*^2^	Primary atom site location: structure-invariant direct methods
Least-squares matrix: full	Secondary atom site location: difference Fourier map
*R*[*F*^2^ > 2σ(*F*^2^)] = 0.027	Hydrogen site location: inferred from neighbouring sites
*wR*(*F*^2^) = 0.075	H atoms treated by a mixture of independent and constrained refinement
*S* = 1.08	*w* = 1/[σ^2^(*F*_o_^2^) + (0.0404*P*)^2^ + 0.7529*P*] where *P* = (*F*_o_^2^ + 2*F*_c_^2^)/3
2028 reflections	(Δ/σ)_max_ = 0.001
108 parameters	Δρ_max_ = 0.57 e Å^−^^3^
2 restraints	Δρ_min_ = −0.32 e Å^−^^3^

### Special details

**Table:**

Geometry. All e.s.d.'s (except the e.s.d. in the dihedral angle between two l.s. planes) are estimated using the full covariance matrix. The cell e.s.d.'s are taken into account individually in the estimation of e.s.d.'s in distances, angles and torsion angles; correlations between e.s.d.'s in cell parameters are only used when they are defined by crystal symmetry. An approximate (isotropic) treatment of cell e.s.d.'s is used for estimating e.s.d.'s involving l.s. planes.
Refinement. Refinement of *F*^2^ against ALL reflections. The weighted *R*-factor *wR* and goodness of fit *S* are based on *F*^2^, conventional *R*-factors *R* are based on *F*, with *F* set to zero for negative *F*^2^. The threshold expression of *F*^2^ > σ(*F*^2^) is used only for calculating *R*-factors(gt) *etc*. and is not relevant to the choice of reflections for refinement. *R*-factors based on *F*^2^ are statistically about twice as large as those based on *F*, and *R*- factors based on ALL data will be even larger.

### Fractional atomic coordinates and isotropic or equivalent isotropic displacement parameters (Å^2^)

**Table:**

	*x*	*y*	*z*	*U*_iso_*/*U*_eq_	
C1	0.59471 (10)	0.18494 (8)	0.0429 (2)	0.0210 (3)	
H1	0.5466	0.1895	−0.0509	0.025*	
C2	0.65854 (10)	0.12686 (8)	0.0035 (2)	0.0236 (3)	
C3	0.72901 (10)	0.12145 (9)	0.1412 (3)	0.0250 (3)	
H3	0.7745	0.0832	0.1193	0.030*	
C4	0.73227 (10)	0.17218 (9)	0.3100 (3)	0.0268 (3)	
H4	0.7800	0.1691	0.4054	0.032*	
C5	0.66527 (9)	0.22760 (9)	0.3387 (2)	0.0224 (3)	
H5	0.6675	0.2617	0.4564	0.027*	
C6	0.65160 (13)	0.07315 (11)	−0.1835 (3)	0.0384 (4)	
H6A	0.6325	0.0196	−0.1399	0.058*	
H6B	0.7094	0.0694	−0.2513	0.058*	
H6C	0.6084	0.0955	−0.2808	0.058*	
Co1	0.5000	0.325574 (15)	0.2500	0.01344 (10)	
N1	0.59730 (8)	0.23485 (7)	0.20607 (18)	0.0175 (2)	
O1	0.59964 (7)	0.41307 (6)	0.21413 (16)	0.0177 (2)	
H1A	0.5893 (14)	0.4584 (10)	0.263 (3)	0.027*	
H1B	0.6013 (12)	0.4199 (11)	0.088 (2)	0.027*	
O2	0.52369 (7)	0.32565 (5)	0.57274 (15)	0.0183 (2)	
O3	0.42370 (6)	0.42849 (6)	0.69346 (15)	0.0181 (2)	
S1	0.5000	0.37749 (3)	0.7500	0.01309 (12)	

### Atomic displacement parameters (Å^2^)

**Table:**

	*U*^11^	*U*^22^	*U*^33^	*U*^12^	*U*^13^	*U*^23^
C1	0.0248 (7)	0.0188 (6)	0.0192 (6)	0.0005 (5)	−0.0001 (5)	−0.0020 (5)
C2	0.0287 (7)	0.0185 (6)	0.0234 (7)	0.0004 (5)	0.0057 (6)	−0.0018 (5)
C3	0.0221 (7)	0.0199 (7)	0.0329 (8)	0.0042 (5)	0.0066 (6)	0.0021 (6)
C4	0.0212 (7)	0.0271 (8)	0.0320 (8)	0.0017 (6)	−0.0033 (6)	0.0009 (6)
C5	0.0222 (7)	0.0236 (7)	0.0215 (7)	−0.0002 (5)	−0.0025 (5)	−0.0027 (5)
C6	0.0487 (10)	0.0320 (9)	0.0346 (9)	0.0125 (8)	0.0008 (8)	−0.0145 (8)
Co1	0.01657 (15)	0.01387 (15)	0.00988 (14)	0.000	0.00000 (8)	0.000
N1	0.0185 (6)	0.0168 (5)	0.0173 (5)	0.0001 (4)	0.0008 (4)	−0.0007 (4)
O1	0.0224 (5)	0.0159 (5)	0.0148 (5)	−0.0010 (4)	−0.0002 (4)	−0.0017 (4)
O2	0.0258 (5)	0.0187 (5)	0.0105 (5)	0.0047 (4)	0.0004 (4)	−0.0012 (3)
O3	0.0200 (5)	0.0184 (5)	0.0159 (4)	0.0032 (4)	−0.0009 (4)	−0.0012 (4)
S1	0.0168 (2)	0.0135 (2)	0.0090 (2)	0.000	0.00065 (14)	0.000

### Geometric parameters (Å, °)

**Table:**

C1—N1	1.3427 (18)	C6—H6C	0.9800
C1—C2	1.392 (2)	Co1—O1	2.1115 (10)
C1—H1	0.9500	Co1—O1^i^	2.1115 (10)
C2—C3	1.391 (2)	Co1—O2^i^	2.1124 (10)
C2—C6	1.506 (2)	Co1—O2	2.1124 (10)
C3—C4	1.380 (2)	Co1—N1	2.1308 (12)
C3—H3	0.9500	Co1—N1^i^	2.1308 (12)
C4—C5	1.385 (2)	O1—H1A	0.835 (15)
C4—H4	0.9500	O1—H1B	0.820 (15)
C5—N1	1.3431 (19)	O2—S1	1.4779 (10)
C5—H5	0.9500	O3—S1	1.4799 (10)
C6—H6A	0.9800	S1—O2^ii^	1.4779 (10)
C6—H6B	0.9800	S1—O3^ii^	1.4799 (10)
			
N1—C1—C2	123.71 (14)	O1^i^—Co1—O2	90.73 (4)
N1—C1—H1	118.1	O2^i^—Co1—O2	179.93 (5)
C2—C1—H1	118.1	O1—Co1—N1	89.05 (5)
C3—C2—C1	117.41 (13)	O1^i^—Co1—N1	177.82 (4)
C3—C2—C6	121.76 (14)	O2^i^—Co1—N1	89.23 (4)
C1—C2—C6	120.82 (14)	O2—Co1—N1	90.82 (4)
C4—C3—C2	119.45 (13)	O1—Co1—N1^i^	177.82 (4)
C4—C3—H3	120.3	O1^i^—Co1—N1^i^	89.05 (5)
C2—C3—H3	120.3	O2^i^—Co1—N1^i^	90.82 (4)
C3—C4—C5	119.27 (15)	O2—Co1—N1^i^	89.23 (4)
C3—C4—H4	120.4	N1—Co1—N1^i^	89.44 (6)
C5—C4—H4	120.4	C1—N1—C5	117.75 (12)
N1—C5—C4	122.40 (14)	C1—N1—Co1	121.65 (10)
N1—C5—H5	118.8	C5—N1—Co1	120.56 (9)
C4—C5—H5	118.8	Co1—O1—H1A	116.7 (15)
C2—C6—H6A	109.5	Co1—O1—H1B	103.1 (13)
C2—C6—H6B	109.5	H1A—O1—H1B	104.9 (17)
H6A—C6—H6B	109.5	S1—O2—Co1	136.19 (6)
C2—C6—H6C	109.5	O2—S1—O2^ii^	108.35 (8)
H6A—C6—H6C	109.5	O2—S1—O3^ii^	109.76 (6)
H6B—C6—H6C	109.5	O2^ii^—S1—O3^ii^	109.58 (5)
O1—Co1—O1^i^	92.51 (6)	O2—S1—O3	109.58 (5)
O1—Co1—O2^i^	90.73 (4)	O2^ii^—S1—O3	109.76 (6)
O1^i^—Co1—O2^i^	89.22 (4)	O3^ii^—S1—O3	109.79 (8)
O1—Co1—O2	89.22 (4)		
			
N1—C1—C2—C3	−0.6 (2)	N1^i^—Co1—N1—C1	65.61 (10)
N1—C1—C2—C6	−179.69 (15)	O1—Co1—N1—C5	61.84 (11)
C1—C2—C3—C4	0.8 (2)	O2^i^—Co1—N1—C5	152.59 (11)
C6—C2—C3—C4	179.90 (15)	O2—Co1—N1—C5	−27.36 (11)
C2—C3—C4—C5	−0.1 (2)	N1^i^—Co1—N1—C5	−116.59 (12)
C3—C4—C5—N1	−1.0 (2)	O1—Co1—O2—S1	78.59 (9)
C2—C1—N1—C5	−0.4 (2)	O1^i^—Co1—O2—S1	−13.91 (9)
C2—C1—N1—Co1	177.45 (11)	N1—Co1—O2—S1	167.62 (9)
C4—C5—N1—C1	1.2 (2)	N1^i^—Co1—O2—S1	−102.95 (10)
C4—C5—N1—Co1	−176.65 (11)	Co1—O2—S1—O2^ii^	138.72 (11)
O1—Co1—N1—C1	−115.96 (11)	Co1—O2—S1—O3^ii^	−101.66 (9)
O2^i^—Co1—N1—C1	−25.22 (11)	Co1—O2—S1—O3	18.98 (11)
O2—Co1—N1—C1	154.83 (11)		

Symmetry codes: (i) −*x*+1, *y*, −*z*+1/2; (ii) −*x*+1, *y*, −*z*+3/2.

### Hydrogen-bond geometry (Å, °)

**Table:**

*D*—H···*A*	*D*—H	H···*A*	*D*···*A*	*D*—H···*A*
O1—H1B···O3^i^	0.82 (2)	1.86 (2)	2.6652 (15)	166.(2)
O1—H1A···O3^iii^	0.84 (2)	1.92 (2)	2.7331 (14)	165.(2)

Symmetry codes: (i) −*x*+1, *y*, −*z*+1/2; (iii) −*x*+1, −*y*+1, −*z*+1.
